# Kinetics and fracture resistance of lithiated silicon nanostructure pairs controlled by their mechanical interaction

**DOI:** 10.1038/ncomms8533

**Published:** 2015-06-26

**Authors:** Seok Woo Lee, Hyun-Wook Lee, Ill Ryu, William D. Nix, Huajian Gao, Yi Cui

**Affiliations:** 1 Geballe Laboratory for Advanced Materials, Stanford University, Stanford, California 94305, USA; 2 Department of Materials Science and Engineering, Stanford University, Stanford, California 94305, USA; 3 School of Engineering, Brown University, Providence, Rhode Island 02912, USA; 4 Stanford Institute for Materials and Energy Sciences, SLAC National Accelerator Laboratory, 2575 Sand Hill Road, Menlo Park, California 94025, USA

## Abstract

Following an explosion of studies of silicon as a negative electrode for Li-ion batteries, the anomalous volumetric changes and fracture of lithiated single Si particles have attracted significant attention in various fields, including mechanics. However, in real batteries, lithiation occurs simultaneously in clusters of Si in a confined medium. Hence, understanding how the individual Si structures interact during lithiation in a closed space is necessary. Here, we demonstrate physical and mechanical interactions of swelling Si structures during lithiation using well-defined Si nanopillar pairs. *Ex situ* SEM and *in situ* TEM studies reveal that compressive stresses change the reaction kinetics so that preferential lithiation occurs at free surfaces when the pillars are mechanically clamped. Such mechanical interactions enhance the fracture resistance of lithiated Si by lessening the tensile stress concentrations in Si structures. This study will contribute to improved design of Si structures at the electrode level for high-performance Li-ion batteries.

## Abstract (web summary)

It is imperative to understand how individual Si nanostructures interact in battery operations. Here, the authors design well-defined crystalline Si nanopillars and show how mechanical interactions of neighbouring Si structures affect their reaction kinetics and fracture resistance during electrochemical lithiation.

## Graphical abstract

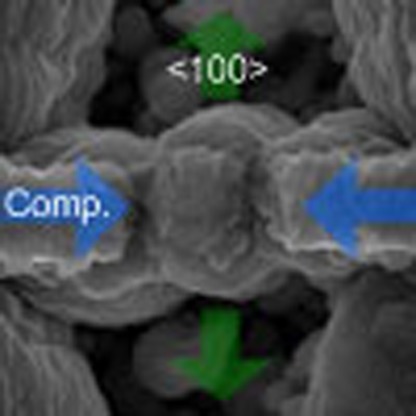

Silicon (Si) has attracted great attention as a promising negative electrode material for Li-ion batteries due to its exceptional theoretical specific capacity of 3,578 mAh g^−1^ for the Li_15_Si_4_ phase at room temperature^1^,^2^,^3^,^4^,^5^. Despite these preeminent theoretical properties, conventional Si anodes face significant challenges due to the large volume changes that accompany lithiation. These effects have limited the choice of Si as a commercial negative electrode because they can lead to the loss of electrical contact between active materials by mechanical fracture, accumulation of solid-electrolyte interphase layers, and rapid capacity fading during electrochemical cycling^6^,^7^,^8^,^9^. Recently, nanotechnology has achieved a breakthrough to overcome the aforementioned challenges of Si as a negative electrode for Li-ion batteries^1^,^2^. Various Si nanomaterials and engineered Si nanostructures such as nanowires/particles, hollow spheres and porous nanostructures have demonstrated stable cycling and resistance to fracture in spite of the large volume change of Si^1^,^10^,^11^,^12^. Engineered nanostructures, wherein the surface of the Si structure does not strain and where a gap for volume expansion of lithiated Si is provided, lead to a stable solid-electrolyte interphase layer formation on the surface of the electrode material and enhanced Coulombic efficiency and markedly improved cycle life^7^,^8^.

Accompanying the search for high-performance Si anodes, fundamental studies have provided a better idea of how Si lithiates, swells and fractures, leading to a basis for the rational design of Si structures^4^. Especially, the extreme volumetric and structural changes of lithiated Si have attracted much attention in mechanics because of the large stress evolution and corresponding mechanical fracture. The marked change of mechanical properties by lithiation has been documented by simulations and experiments^13^,^14^,^15^,^16^,^17^,^18^. Analytical and numerical analyses, including both elasticity and plasticity, have suggested both diffusion-induced stress models and pressurized hollow structure models of lithiation/delithiation of Si as a part of an effort to explain how the expansion causes stress evolution and mechanical fracture^19^,^20^,^21^,^22^,^23^,^24^. These models are based on experimental observations such as volumetric changes, mechanical fracture and structural changes^25^,^26^,^27^,^28^. Recently, top–down fabrication of Si nanostructures allowed the systematic study of the effects of crystal orientation, dimensions and morphology that revealed preferential lithiation along <110> directions of crystalline Si, a size dependence of the fracture resistance, and the robustness of amorphous Si^6^,^29^,^30^,^31^,^32^,^33^. *In situ* transmission electron microscopy (TEM) has provided time-series crystallographic and chemical information as well as information about the morphology of lithiated Si^34^. The observed dynamic behaviour of Si nanostructures provided information about the kinetics of lithiation controlled by mechanical stresses and the orientation of the reaction interface of crystalline Si as well as the aforementioned anisotropic expansion and fracture behaviour^35^,^36^,^37^,^38^,^39^.

However, in a real battery system, Si structures form as clusters at the electrode level and the lithiation of the individual structures occurs simultaneously in a confined medium. Then, swelling Si structures in fixed volume mechanically interact with each other and the reaction kinetics and fracture behaviour become more complicated than that observed for single-particle systems. Therefore, understanding how the individual Si structures mechanically interact during lithiation is necessary for the rational design of Si electrodes. Here we show how mechanical interactions of neighbouring crystalline Si structures affect their reaction kinetics and fracture resistance during electrochemical lithiation, using *ex situ* scanning electron microscopy (SEM) and *in situ* TEM of Si nanopillar pairs.

## Results

### Lithiation of mechanically clamped Si pillar

To mimic the cluster of crystalline Si particles in the confined volume in the negative electrode of a Li-ion battery, Si nanopillars with adjacent rigid walls were fabricated by e-beam lithography and dry etching of <110> single crystalline Si wafer (see Methods and Supplementary Fig. 1a–c). We used <110> Si pillars so that lateral volume expansion would occur preferentially along two opposite <110> directions on lithiation. To simulate mechanical clamping of Si structure in closed-packed media, e-beam lithography defined the various diameters of the pillars and the location of rigid walls for two different geometries so that rigid walls block both sides of <110> direction of the pillar. For the *ex situ* SEM study, the fabricated silicon nanopillar and wall array on a piece of wafer was lithiated by sweeping voltage down to 10 mV versus Li/Li^+^ and held for more than 10 h in a half cell with Li foil (see Methods and Supplementary Fig. 1d–i). For the *in situ* TEM study, the pillars were placed at the edge <110> direction of the piece of <110> wafer and mounted on the TEM holder with a proper tilting so that the pillar can be observed under e-beam without shading (see Supplementary Fig. 2). After building the solid cell configuration with a Li/Li_2_O counter electrode, the pillar is lithiated by applying d.c. bias during the TEM observation.

**Figure 1:**
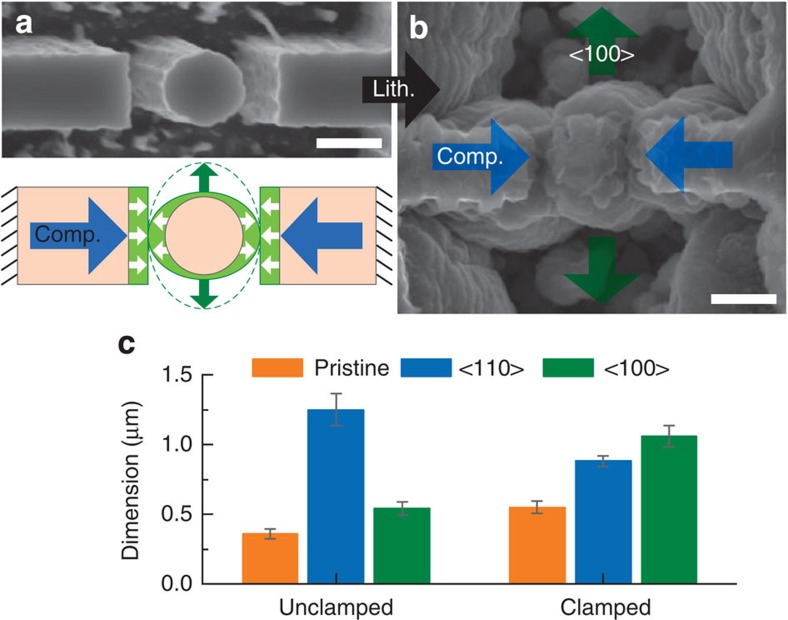
SEM study of the lithiation of a clamped <110> Si nanopillar. (**a**,**b**) SEM images of <110> Si nanopillar positioned between adjacent rigid walls before (**a**) and after (**b**) lithiation. The electrochemical lithiation of a single pillar was suppressed by compressive stresses between the two rigid walls, which were supposed to be preferably grown to <110> direction as displayed in a schematic diagram (**a**). (**c**) Column chart of dimension change of <110> Si nanopillar along <110> (blue) and <100> (green) direction after lithiation when the pillar is unclamped^30^ and clamped. Single <110> Si nanopillar standing alone has preferential lithiation along <110> directions of Si but the clamped Si nanopillar shows further expansion along <100> direction.

To simulate the mechanical clamping of Si structures in closed-packed media, a pillar was prepared between two rigid walls blocking both <110> directions on lithiation as shown in Fig. 1. A <110> Si pillar 550 nm in diameter standing between two rigid walls with 320 nm gaps was fabricated for the SEM study (Fig. 1a). Since the crystal orientation is identical to the first case, the pillar and the walls expand laterally along <110> directions and fill the gap between them on lithiation. After the contact, the lithiation along the <110> direction cannot proceed due to the build-up of compressive stresses and the pillar lithiates along a second favoured direction, <100> as shown in Fig. 1b. The walls also expand along the <100> direction after contact with the pillar. Figure 1c compares dimension changes of the unclamped and clamped <110> pillars. The unclamped pillar with a diameter of 0.36 μm expands to 1.25 and 0.54 μm along <110> and <100> directions, respectively, on lithiation, as found in our previous study^30^. The unclamped pillar clearly shows anisotropic expansion behaviour where the <110> direction exhibits a faster reaction than the <100> direction. In contrast, the clamped pillar with a diameter of 0.55 μm expands to 0.88 and 1.06 μm along the <110> and <100> directions, respectively, on lithiation. Ideally, the swelling pillar and wall would come into contact in the middle of the gap and the width of the pillar along the <110> direction would then be 0.87 μm (=original diameter+2 × gap/2), which is indeed very close to the measured width. Therefore, it is clear that the lithiation along the <110> direction is stopped at the point of contact and the subsequent lithiation continues along the <100> direction.

**Figure 2:**
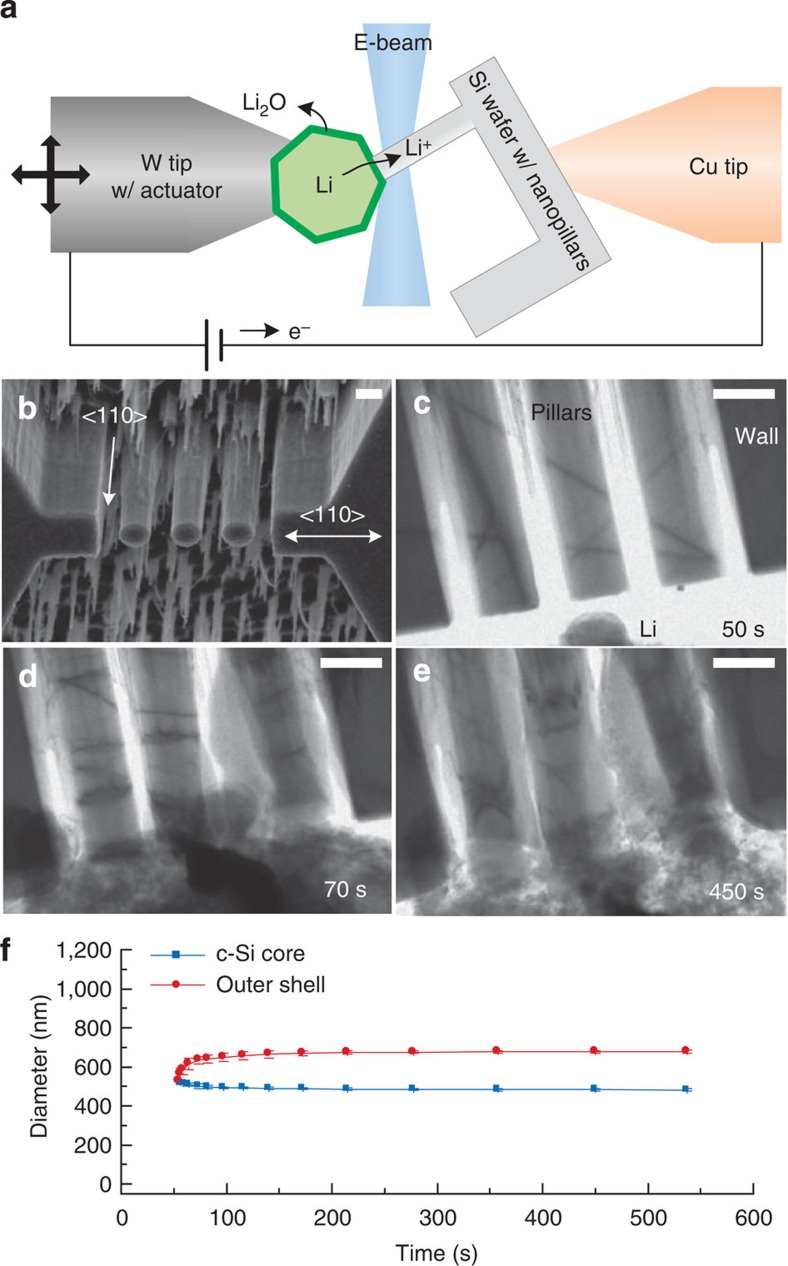
*In situ* TEM study of the lithiation of a clamped <110> Si nanopillar. (**a**) A schematic image of the electrochemical cell configuration for *in situ* TEM observation. E-beam penetrates through <100> direction of Si nanopillar to observe a lateral <110> expansion during lithiation. (**b**) SEM image of pristine three pillars with adjacent rigid walls on both sides for in situ TEM observation. (**c**–**e**) Time series of TEM images of the pillars during lithiation. All scale bars in SEM and TEM images are 500 nm. (**f**) The diameters of crystalline Si core and lithiated outer Li_*x*_Si for the time line in the middle of lithiation. The lithiation cannot proceed further along <110> direction against the neighboring pillars due to the mechanical clamping.

*In situ* TEM observation of the <110> Si pillar near the wall provides a better picture of the dynamic lithiation behaviour of the crystalline Si core and the corresponding mechanical interaction. The electron beam penetrates through the <100> direction of the <110> Si nanopillar standing by the rigid wall, so a lateral <110> expansion of the nanopillar can be monitored during the lithiation process (Fig. 2a,b). The reaction stoppage of the pillar after the contact is clearly shown in the *in situ* TEM study. For the mechanical clamping, three <110> Si nanopillars with the same diameters of 550 nm and rigid walls on either side of the pillars were fabricated as shown in Fig. 2b. A single pillar clamped by two rigid walls also exhibits termination of the expansion as shown in *ex situ* SEM, but overlapping of structures hindered precise measurement (see Supplementary Movie 1). The time series of TEM images of the lithiating pillars clamped by the walls are shown in Fig. 2c–e (see also Supplementary Movies 2 and 3). At the beginning of the lithiation, the pillars start to expand as a normal <110> single pillar does in spite of inconsistent expansion due to irregular contact with Li metal (Fig. 2c,d). After the contact, a Li_*x*_Si shell fills the empty space and the crystalline Si core stops shrinking due to the termination of the lithiation (Fig. 2e). The plot of the diameters of the Li_*x*_Si outer shell and the crystalline Si core as a function of time clearly shows that the expansion of the shell and shrinkage of the core are slowing down on the contact and halted at about 90 s (Fig. 2f). Since then, the diameter of the remaining crystalline core is maintained for over 400 s and the lithiation cannot proceed further along <110> direction against the neighbouring pillars due to the mechanical clamping. In contrast, unclamped pillar exhibits the completed lithiation and the mechanical fracture without the termination of the lithiation (see Supplementary Movies 4 and 5).

### Analytical model

**Figure 3:**
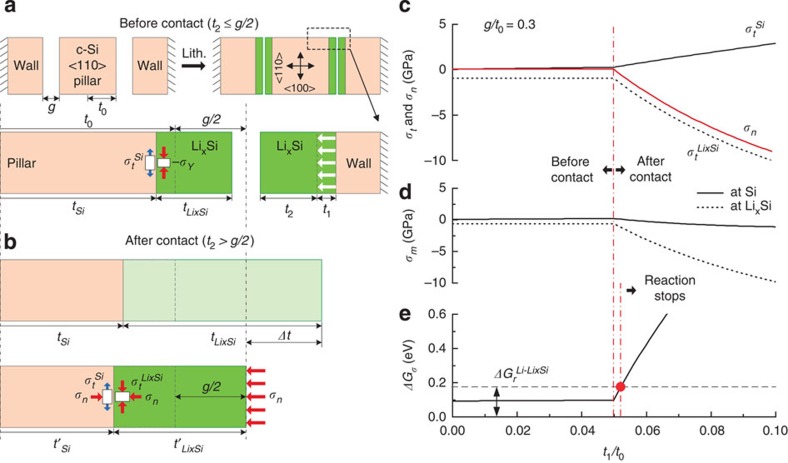
Analytical model of the clamped Si pillar to predict the change of the driving force of the reaction. (**a**) A schematic view of <110> crystalline Si with wall fixed at the end. The scheme represents morphological expansion and induced stresses during lithiation of <110> pillars and walls before the physical contact (‘Before contact', *t*_2_*<g/*2). (**b**) A schematic view of the one side of Si pillar contacted with the wall physically (‘After contact', *t*_2_*≥g/*2). The displacement of lithiated Si is confined as a half of the gap (*g/*2). (**c**) Normal (*σ*_n_) and tangential (*σ*_t_) stress at the interfaces in the crystalline Si and Li_*x*_Si for the depth of lithiation (*t*_1_*/t*_0_) when *g/t*_0_ is 0.3. (**d**) Mean stress (*σ*_m_) at the interfaces in the crystalline Si (solid) and Li_*x*_Si (dotted) for the depth of lithiation (*t*_1_*/t*_0_) when *g/t*_0_ is 0.3. (**e**) Corresponding change of free energy due to mechanical stress (Δ*G*_*σ*_) for the depth of lithiation (*t*_1_*/t*_0_) when *g/t*_0_ is 0.3. Black dash line represents free energy of Li deposition versus free energy of lithiation of Si (
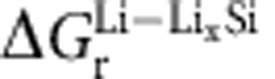
). Red vertical lines indicate the contact and reaction stoppage on lithiation of Si, respectively.

To explain how mechanical clamping stops the lithiation at the contact, an analytical model is developed by considering mechanical stress evolution on the clamping and change of driving force of the reaction. The driving force of the lithiation is defined as:


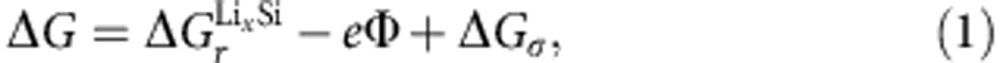


where Δ*G* is the change of Gibbs free energy, 
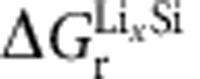
 is the change of free energy of lithiation without applied voltage or mechanical stress, Φ is the applied voltage to the electrochemical cell, and Δ*G*_*σ*_ is the change of free energy due to mechanical stress^21^. Δ*G*_*σ*_ expresses the relationship between mechanical stress at the atomically sharp interface of crystalline Si and swelling Li_*x*_Si alloy and the change of the driving force of the reaction^28^. Considering the consumption of one Li atom to form 1/*x* units of Li_*x*_Si, Δ*G*_*σ*_ is computed as^21^,^35^:


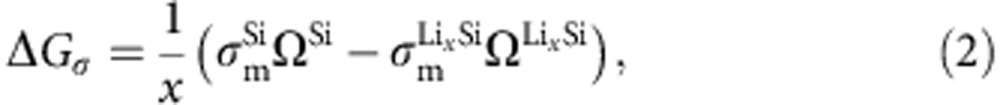


where *σ*_m_^Si^ and 
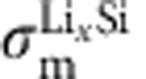
 are the mean stresses in the crystalline Si and in the Li_*x*_Si at the interface, respectively, and Ω^Si^ and 
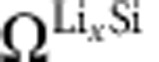
 are the volumes per Si atom and unit of Li_*x*_Si, respectively. Since a negative Δ*G* drives lithiation, compressive hydrostatic stress in the crystalline Si or tensile hydrostatic stress in the Li_*x*_Si enhances lithiation process. The model for the estimation of stress on lithiation includes consideration of both the ‘Before contact' and ‘After contact' of neighbouring Si structures. Figure 3a shows a schematic view of the model of ‘Before contact'. A square <110> Si pillar of *2t*_*0*_ width is located between two fixed rigid-wall structures aligned along the lateral <110> direction with a gap of *g*. Assuming dominant expansion and propagation of flat {110} interface along the <110> direction as shown in the previous studies^31^, after lithiation the thicknesses of crystalline Si core and the Li_*x*_Si layer may be called *t*_Si_ and 
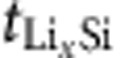
, respectively. Also *t*_1_ (
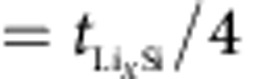
) is the thickness of the consumed crystalline Si and *t*_2_ is the displacement of each surface towards each other. Before the structures contact each other, Li_*x*_Si in the gap is free to expand laterally along the <110> direction, which is normal to the interface, so the normal stress (*σ*_n_) is zero. The tangential biaxial stress at the interface in Li_*x*_Si (
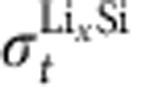
) is equal to the compressive yield strength (*−σ*_*Y*_) assuming plastic deformation in the lithiated Si^21^. Before the contact, mechanical equilibrium requires the tangential biaxial stress at the interface in crystalline Si (*σ*_*t*_^Si^) to be related to 
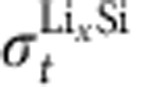
 and the ratio of the thickness of Li_*x*_Si (
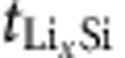
) to the half thickness of crystalline Si (*t*_Si_), as follows:


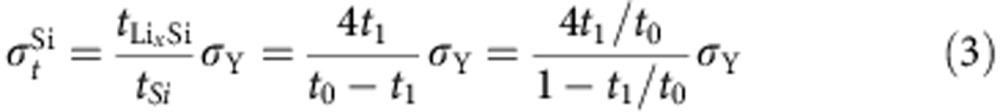


Then, the mean stresses at the interfaces in the crystalline Si core and in the Li_*x*_Si layer are expressed as:

Before contact:


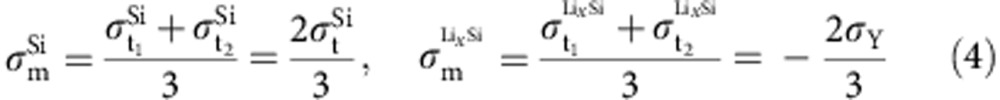


where 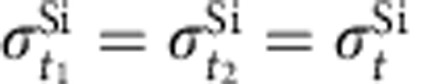 and 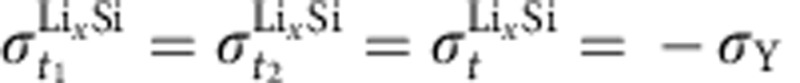. After contact, the displacement of each of the two surfaces is limited to half of the initial gap, *g/*2 and a normal stress at the interface, *σ*_n_ develops on the {110} interface in crystalline Si and on the Li_*x*_Si layer (Fig. 3b). Since the deformation is fully constrained by the contact and the interfacial compatibility, additional plastic deformation is no longer possible and additional lithiation-induced strain must be accommodated by the elastic deformation. In this case, the stress state in the Si core and Li_*x*_Si layer can be computed by the superposition of the normal stress. The tangential stress at the interface in Li_*x*_Si is then determined from the von Mises yield criterion, as follows:


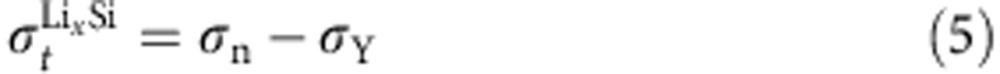


From the force equilibrium and displacement constraint from the gap, the tangential stress at the interface in crystalline Si is then expressed as:


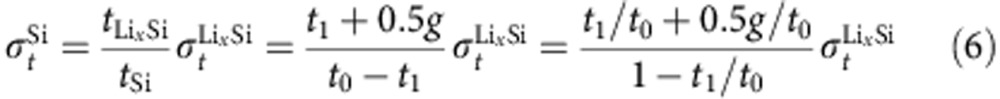


Then, the mean stresses in crystalline Si and Li_*x*_Si at the interface would be given as:

After contact, {110}:


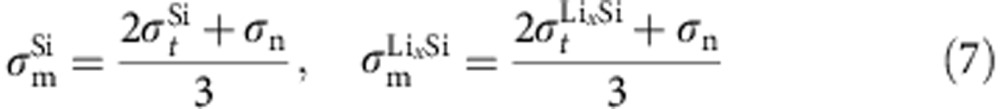


where, as shown below, *σ*_n_ is a negative quantity. Considering the limitation that the surface displacement due to swelling of Li_*x*_Si along the <110> direction is equal to half of the initial gap, *g/*2 and assuming that elastic deformation accommodates further growth of the layer and that lateral flow of Li_*x*_Si is suppressed, the normal stress (*σ*_n_) that develops after contact as a function of the extent of continued lithiation may be estimated using a simple uniaxial stress analysis. For this analysis the dimension *t*_1_, the thickness of the consumed Si layer, may be taken as a measure of the extent of lithiation. As shown in the Supplementary Note 1, the axial stress that develops after contact can be calculated as:


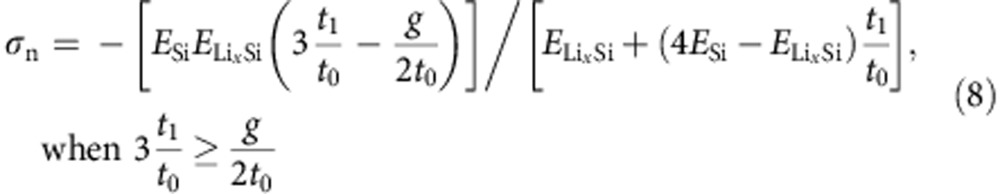


where *E*_Si_ and *E*_Li*x*Si_ are Young's modulus of crystalline Si and Li_*x*_Si, respectively.

In the estimation of the stress, the considered yield strength of Li_*x*_Si (*σ*_*Y*_) is 1.0 GPa, and *E*_Si_ and 
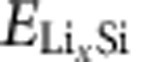
 are 180 and 35 GPa, respectively^15^,^16^. The ratio of the gap and initial thickness of crystalline Si (*g/t*_0_) are 0.3, 0.6, 1.2, and 2.4. Figure 3c–e show tangential, normal, and mean stresses and the change of free energy due to mechanical stress versus the extent of lithiation (*t*_1_*/t*_0_) when *g/t*_0_ is 0.3 (see also Supplementary Fig. 3a–d). The normal stress (*σ*_n_) acting in both crystalline Si and Li_*x*_Si rapidly becomes more compressive after the contact (red solid line in Fig. 3c). The compressive tangential stress at the interface in Li_*x*_Si (
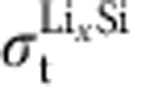
) develops after the contact together with the compressive normal stress (dashed line in Fig. 3c). The tangential stress at the interface in crystalline Si (*σ*_t_^Si^) rapidly increases after the contact as the tangential stress at the interface in Li_*x*_Si decreases (black solid line in Fig. 3c). The mean stresses at the interfaces in crystalline Si and Li_*x*_Si (*σ*_m_^Si^ and 
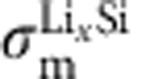
) for a given extent of lithiation (*t*_1_*/t*_0_) are calculated by equations (3, 4, 5, 6, 7, 8) as shown in Fig. 3d. *σ*_m_^Li*_x_*Si^ is constant before contact and *σ*_m_^Si^ increases slightly due to the increase of the tangential stress in crystalline Si on lithiation (see also Supplementary Fig. 3c). After contact, 
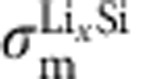
 becomes more compressive following the trend of the normal stress. Assuming that 
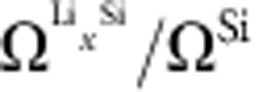
 is 4 and *x* is 3.75 (Li_3.75_Si) considering a 400% volume change for fully lithiated Si at room temperature, Figure 3e shows the change of free energy due to mechanical stress at the interface (Δ*G*_*σ*_) for the extents of lithiation (*t*_1_*/t*_0_) corresponding to the mean stresses shown and explains how mechanical clamping along the <110> direction suppresses the lithiation of crystalline Si at the interface. Before the contact, Δ*G*_*σ*_ slightly increases from 0.09 to 0.094 eV and the lithiation along <110> direction is continued spontaneously since the free energy of Li deposition versus lithiation of Si (
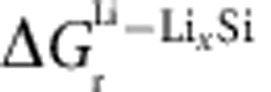
) is 0.18 eV^40^. After the contact, as *σ*_n_ and *σ*_*t*_^Li*x*Si^ become more compressive, the increasing Δ*G*_*σ*_ reduces the gap of the net driving force between the lithiation of Si and Li deposition. Then, finally, Δ*G*_*σ*_ exceeds 
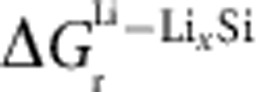
 as marked as a red dot in Fig. 3e and lithiation of Si is stopped at the interface where the physical interaction induces a sufficiently big compressive normal stress (<110> direction in the experiment). After this point is reached Si is lithiated mainly along the other direction, free from the physical contact (<100> direction in the experiment). From the point of contact to the point at which the reaction is stopped the extent of lithiation (*t*_1_*/t*_0_) changes by only 0.0025 (red dashed line), which means that mechanical contact can effectively prohibit further lithiation right after the contact is made. For larger gaps, the lithiation after contact goes further but is still less than 0.4% (see Supplementary Fig. 3d).

### Mechanical fracture

**Figure 4:**
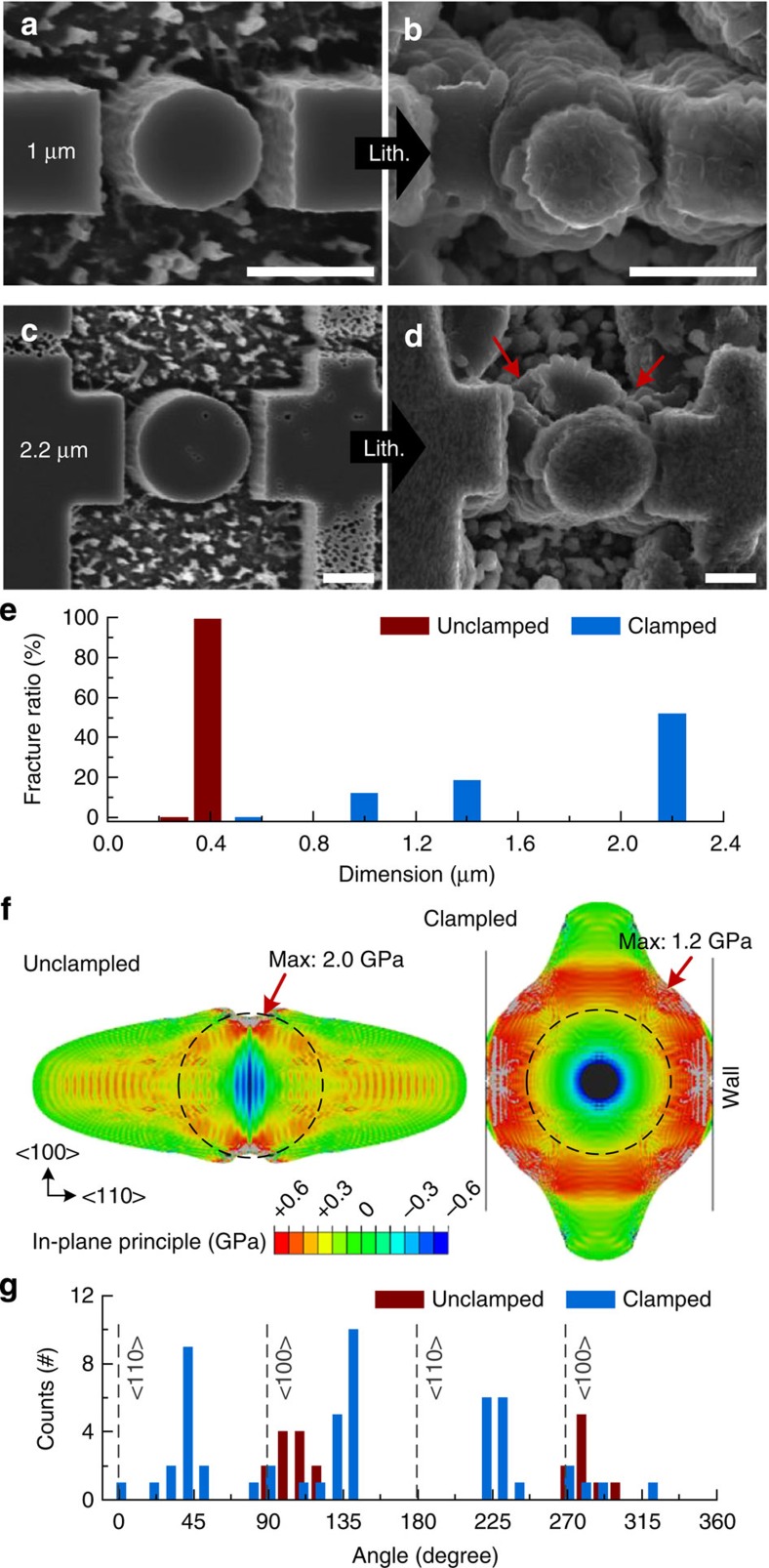
Improved fracture resistance of the clamped Si nanopillar on lithiation. (**a**,**b**) SEM images of crystalline <110> Si pillar of 1-μm diameter and the walls with gap of 300 nm. The pillar is clamped by the walls and expanded along <100> direction upon lithiation. Significant crack is not found. (**c**,**d**) SEM images of crystalline <110> Si pillar of 2.2 μm diameter and the walls with gap of 300 nm. After lithiation, the cracks are found between <110> and <100> directions as indicated by red arrows. Scale bars, 1 μm. (**e**) Column chart of the fracture ratio of the clamped <110> Si pillars for various diameters. To compare the effect of mechanical clamping for the fracture resistance, the fracture ratio of unclamped <110> pillar is shown as red columns^20^. (**f**) Finite element analysis of in-plane principal stress of unclamped (left) and clamped (right) <110> Si pillar after full lithiation. Initial diameter is 550 nm (dot circle) and lateral displacement of clamped pillar is confined to 160 nm (solid line). (**g**) Column chart of the population of the fracture location as an angle of the crack in the clamped <110> Si pillar upon lithiation (blue). The population of the fracture location of the unclamped <110> pillar (red) compares how mechanical clamping changes the fracture behaviour^6^.

Mechanical clamping of a Si structure on lithiation affects the fracture behaviour as well as the preferred direction of lithiation. Figure 4 shows how mechanical clamping enhances the fracture resistance of the lithiated Si pillar. The unclamped Si pillar has a critical diameter of ∼300 nm for fracture and the fracture ratio is almost 100% when its diameter is >300 nm^6^. However, the clamped <110> Si pillar with a diameter of 1 μm and a gap of 300 nm expands along the unclamped <100> directions and only a few pillars show noticeable cracking after lithiation (Fig. 4a,b). But the clamped pillar shows size dependent fracture on lithiation just as the unclamped pillar does. When the diameter of the pillars increases to 2.2 μm with a 300 nm gap, the pillars still show expansion along <100> direction but then significant cracks are found between <110> and <100> directions (Fig. 4c,d). The statistical study of fracture ratio of the pillars can clearly show different fracture resistance for clamped and unclamped cases (Fig. 4e). The fracture ratio is obtained by counting the number of fractured pillars with various diameters (see Supplementary Fig. 4). The unclamped pillar shows a significant increase of fracture ratio from 0 to 99% when the diameter increases from 0.26 to 0.39 μm, as reported in our previous study^20^. In contrast, here the fracture ratio is 0% when the diameter of the clamped pillar is 0.55 μm and only gradually increases as the diameter increases. When the diameters of clamped pillars are 1, 1.4, and 2.2 μm, the fracture ratios are 12%, 19%, and 52%, respectively. The diameter of the largest pillar in the test is about seven times of critical diameter of the unclamped pillar for fracture, but half of them have not fractured.

A finite element analysis can be used to explain how mechanical clamping affects the stress distribution and enhances fracture resistance of the Si pillar on lithiation. For this analysis the initial diameter of the simulated <110> Si pillar is 550 nm (dashed circle) and the gap between the pillar and the wall is 160 nm (see Fig. 4f). For the lithiation, the artificial moving boundaries between crystalline Si and Li_*x*_Si have a marching speed ratio of 5:1 along <110> and <100> directions, respectively, as in our previous analysis^30^ (see Supplementary Fig. 5). For the clamped pillar, the movement of the interface along the <110> direction is forced to stop after full contact is made (contact area does not increase). The volume change of lithiated Si is 400% and the considered mechanical properties are same with the analysis above (see Supplementary Note 2 and Supplementary Table 1). Figure 4f compares the estimated in-plane principal stress of a fully lithiated Si pillar with/without mechanical clamping. As our previous studies have shown, the unclamped pillar shows a concentration of tensile stress as high as 2 GPa on the top and bottom of the pillar along the <100> direction^20^. The clamped pillar shows the concentration of tensile stress on the surface of the pillar along the diagonal direction between <110> and <100> after the contact with the wall (see also Supplementary Movies 6 and 7). But the maximum tensile stress for the clamped pillar is only as high as 1.2 GPa. The lower maximum tensile stress for the clamped pillar compared with that for the unclamped pillar is caused by the compressive stresses associated with mechanical clamping, which leads to an enhanced fracture resistance, as shown in the experiment (Fig. 4e). The mechanical clamping also changes the fracture location. The statistical study of the population of crack locations on the pillar (Fig. 4f) shows that the favoured fracture site of the clamped pillar is located along a diagonal between the <110> and <100> directions (see Supplementary Fig. 6).

## Discussion

Fundamental studies of Si as a negative electrode material for electrochemical reactions with Li have revealed how the mechanical stress caused by the large volume changes associated with the reaction plays an important role in both control of the reaction and fracture of the Si structures. However, while most studies have focused on the mechanical behaviour of individual Si particles, wires or pillars, Si anodes in batteries are composed of clusters of particles or wires of different shapes all in a confined space. In the present work, *ex situ* SEM and *in situ* TEM techniques were used to study the effects of mechanical interactions of well-defined crystalline Si nanopillar pairs during lithiation and how those interactions affect both the reaction kinetics and the fracture behaviour. When the Si structure is mechanically clamped by adjacent rigid walls along <110> directions, the reaction in that direction is suppressed by compressive stresses that reduce the driving force for lithiation in that direction. This causes lithiation to occur in the transverse, <100>, direction which is not favoured for unconstrained particles, wires or pillars. On the basis of our observations, we can imagine that the overall lithiation behaviour of real electrodes involve the swelling Si particles that push each other and translate to empty space until clamped conditions are reached. After the clamping of the most favoured lithiation directions, the reactions at the contact points are suppressed by compressive stresses and the other directions free from the clamping are consequently lithiated, much like filling the empty space (see Supplementary Fig. 7). Mechanical clamping of lithiated Si also markedly enhances the fracture resistance and increases the critical size for fracture because compressive stresses at the contact point compensate the concentrated tensile stress at the free surface. Thus, we can anticipate that the Si particles in the clusters in Li-ion batteries become more resistant to fracture than the individual Si structures that have received most attention. Although compressive stresses enhance the fracture resistance and promote filling of the empty space in the Si particle clusters, a space considering 400% volume change to allow complete lithiation is required to use maximum charge capacity of Si anode. Hence, further investigation is necessary to optimize the particle size and the empty space preventing mechanical fracture as well as allowing complete lithiation. In addition, since pristine crystalline Si remains amorphous after the first lithiation, the study of mechanical interaction of amorphous Si during electrochemical reaction is also demanded. Nevertheless, we believe that this study of mechanical interaction of lithiated Si pillars provides better idea of how Si structure will be studied and designed in the electrode level for high-performance Li-ion batteries.

## Methods

### Fabrication of Si nanopillar

<110> crystalline Si pillar with walls was fabricated by e-beam lithography and dry etching (see Supplementary Fig. 1). Poly(methyl methacrylate) (PMMA) pattern for the mask of dry etching was defined on <110> single crystalline Si wafer by e-beam lithography (Nova NanoSEM 450 Scanning Electron Microscope, FEI). Then, the Si wafer is etched by deep reactive ion etching (Deep RIE) process for 10∼15 min with SF_6_ gas for etching and C_4_H_8_ gas for passivation (Surface Technology Systems Co.). Finally, acetone and methanol cleaning removed PMMA pattern on the etched Si pillar and walls. For *in situ* TEM study, Si wafer was cut along <110> direction by K&S 775 Wafer Dicing Saw and PMMA pattern was defined on the cutting edge of the wafer (see Supplementary Fig. 2). The last fabrication process was same as mentioned above.

### Electrochemical characterization by use of *ex situ* SEM

A piece of Si wafer with the pillar and wall structures as a working electrode was assembled with a polymer separator (Nagase & Co. Ltd) and Li metal foil as a counter and reference electrode to build a sandwich structure of a half cell (see Supplementary Fig. 1i). BioLogic VMP3 multichannel battery tester-swept voltage of the cell down to 10 mV versus Li/Li^+^ with a scan rate of 0.1 mV s^−1^ and it is was held for >10 h for complete lithiation of the pillars. After the lithiation, the cell was disassembled and the electrode containing lithiated pillars was washed with acetonitrile to remove residual electrolyte in Ar-filled glove box. The sample was sealed in a vial in the glove box to avoid the oxidation of the sample and transferred to the vacuum chamber in SEM within 15 s.

### *In situ* TEM observation

The *in situ* electrochemical test was carried out in an FEI Titan 80–300 environmental TEM at the acceleration voltage of 300 kV. Nanofactory Instruments Dual-Probe STM–TEM *in situ* sample holder was employed to apply bias between Si nanopillars and Li metal counter electrode. During transferring the Li metal electrode inside TEM, the electrode was exposed to air for about 5 s to create a thin Li_2_O layer of about 20 nm functioning as a solid electrolyte. A relative bias of −4 or −5 V was applied between the two electrodes, which caused Li^+^ ions to be transferred to Si nanopillar electrode through the electrolyte.

## Additional information

**How to cite this article:** Lee, S.W. *et al.* Kinetics and fracture resistance of lithiated silicon nanostructure pairs controlled by their mechanical interaction. *Nat. Commun.* 6:7533 doi: 10.1038/ncomms8533 (2015).

## Supplementary Material

Supplementary Information Supplementary Figures 1-7, Supplementary Table 1, Supplementary Notes 1-2 and Supplementary References

Supplementary Movie 1 Lithiation of a single Si nanopillar between two neighboring rigid walls. The lithiated Si of both nanopillar and the walls overlapped during electrochemical reaction showing undistinguishable area between core and shell. The movie is played at 60x speed.

Supplementary Movie 2 Lithiation of three Si nanopillars between neighboring rigid walls. After the pillars contact each other, the lithiation process cannot proceed further against the neighboring pillars. The movie is played at 30x speed.

Supplementary Movie 3 Another lithiation of three Si nanopillars between neighboring rigid walls. As shown in Movie 2, the termination of lithiation occurred in a similar way. The movie is played at 25x speed.

Supplementary Movie 4 Lithiation of two Si nanopillars without neighboring rigid walls. The expansion and fracture of the two pillars proceeded with the crystalline core transition. The movie is played at 15x speed.

Supplementary Movie 5 Lithiation of a single Si nanopillar with a neighboring rigid wall. The expansion of the LixSi proceeded until the fracture of the pillar occurred to the center of the crystalline core referred to the <100> direction. Translation of the pillar occurred against the interaction of the wall. The movie is played at 20x speed.

Supplementary Movie 6 Numerical analysis of lithiation of unclamped <110> Si nanopillars without neighboring rigid walls. Li concentration (left) and in-plane principle stress (right) of unclamped <110> Si pillar of 550 nm initial diameter are shown as a color map upon lithiation when artificial moving interface of crystalline Si and LixSi is given.

Supplementary Movie 7 Numerical analysis of lithiation of clamped <110> Si nanopillars with neighboring rigid walls. Li concentration (left) and in-plane principle stress (right) of clamped <110> Si pillar of 550 nm initial diameter are shown as a color map upon lithiation when artificial moving interface of crystalline Si and LixSi is given. The initial gap between the pillar and the wall is 160nm.
