# Supplementary material for: Kinetics and fracture resistance of lithiated silicon nanostructure pairs controlled by their mechanical interaction
Source: Nat Commun. 2015 Jun 26;6:7533. doi: 10.1038/ncomms8533 (PMC4491816; doi:10.1038/ncomms8533)
Supplement: Supplementary Information — Supplementary Figures 1-7, Supplementary Table 1, Supplementary Notes 1-2 and Supplementary References [file ncomms8533-s1.pdf]

Supplementary Figures

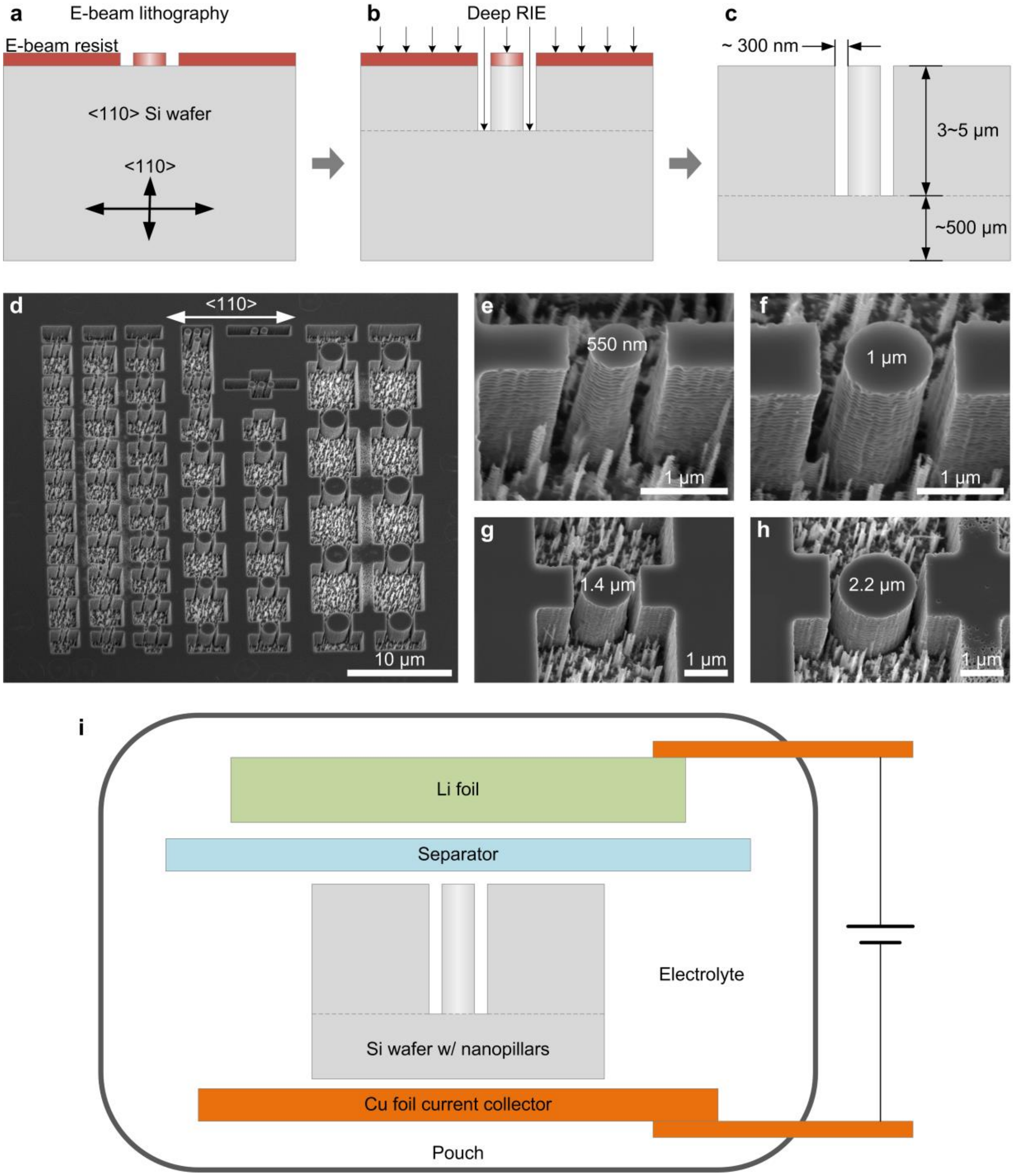

Supplementary Figure 1 | Fabrication of Si nanopillars for *ex situ* SEM study. a-c, Schematic view of fabrication steps of Si nanopillars on <110> Si wafer. **d-h,** SEM images of

fabricated Si pillars with wall structures after deep reactive ion etching for 10~15 min. The diameter of the pillar is varied from 0.55 to 2.2 μm. Scale bars are 10 μm for **(d)** and 1 μm for the others. **i,** Schematic view of a half-cell of the Si pillar and Li metal foil in Al pouch sealed in Ar glove box.

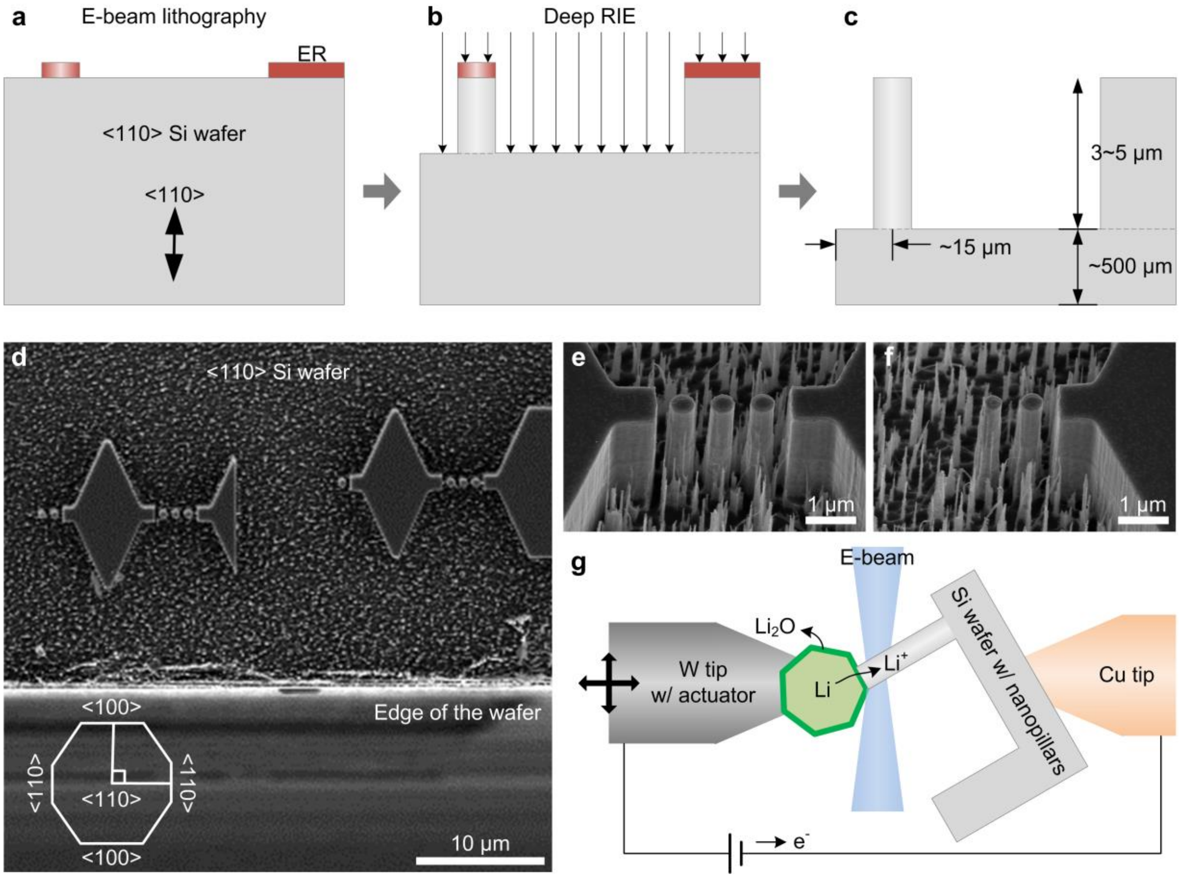

Supplementary Figure 2 | Fabrication of Si nanopillars for *in situ* TEM study. a-c, Schematic view of fabrication steps of Si nanopillars on the edge of the <110> Si wafer. **d-f,** SEM images of fabricated <110> Si pillars with wall structures aligned along <110> direction. Scale bars are 10 μm for (d) and 1 μm for the others. **g,** Schematic view of *in situ* TEM setup with the pillar on the edge of the wafer and Li metal with Li_2O coating.

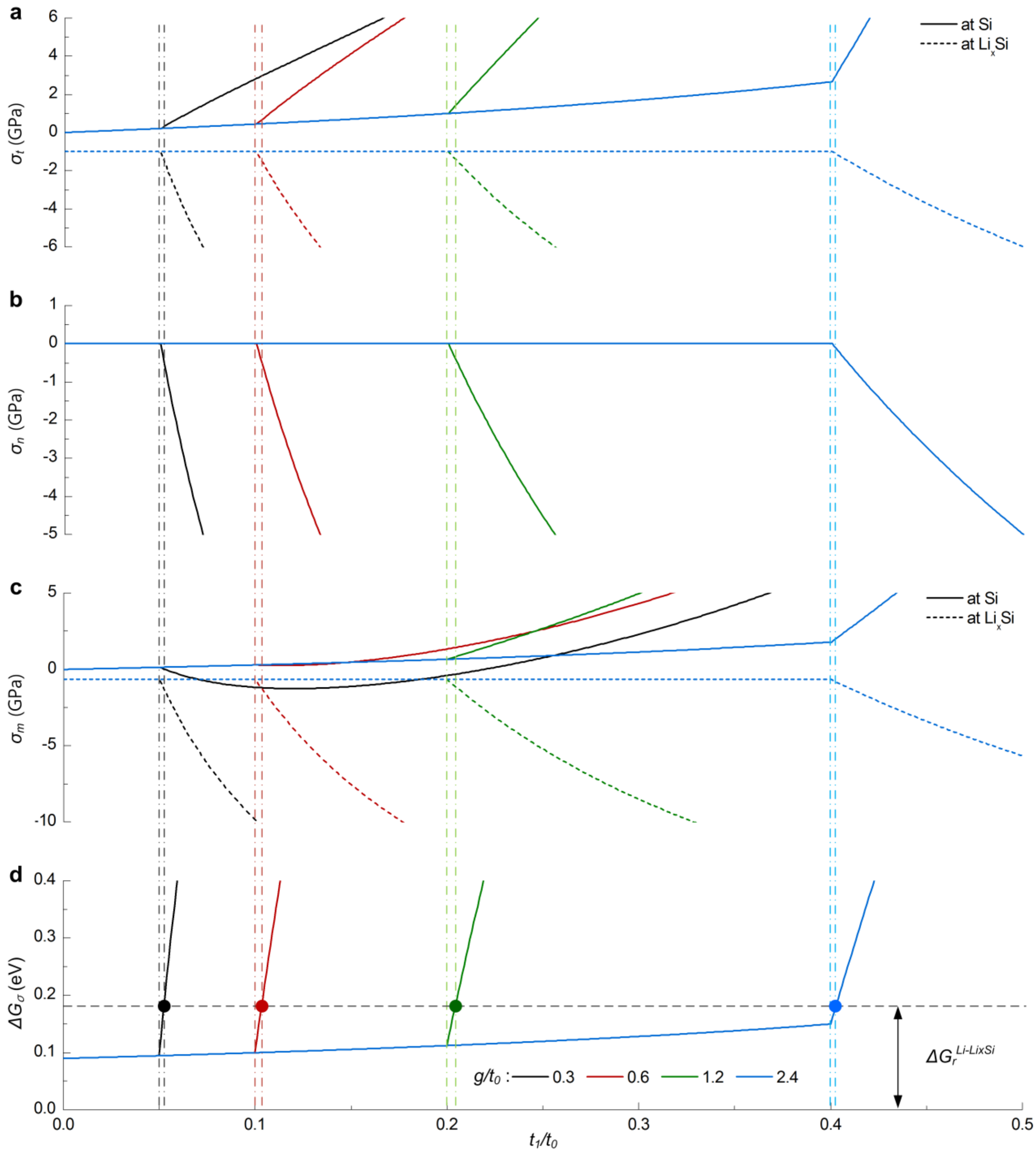

Supplementary Figure 3 | Analysis of mechanical stress of clamped Si structure. a, Tangential stress at the interface for the depth of lithiation (t_1/t_0) when the ratio between the gap and initial thickness of pristine Si (g/t_0) are varied from 0.3 to 2.4. **b,** Normal stress (σ_n) becomes more compressive as the lithiation goes on after the contact. The vertical dash dotted lines

indicate the contact for each g/t_0. **c**, Mean stress in crystalline Si (σ_m^{Si}, solid) and Li_xSi (σ_m^{LixSi} , dotted) at the interface when g/t_0 are varied from 0.3 to 2.4. **d**, Corresponding change of free energy due to mechanical stress (ΔG_σ) for the depth of lithiation (t_1/t_0) when g/t_0 are varied from 0.3 to 2.4. Black dash line represents free energy of Li deposition versus free energy of lithiation of Si ($\Delta G_r^{Li\text{-}LixSi}$ ≈0.18 eV)[1].

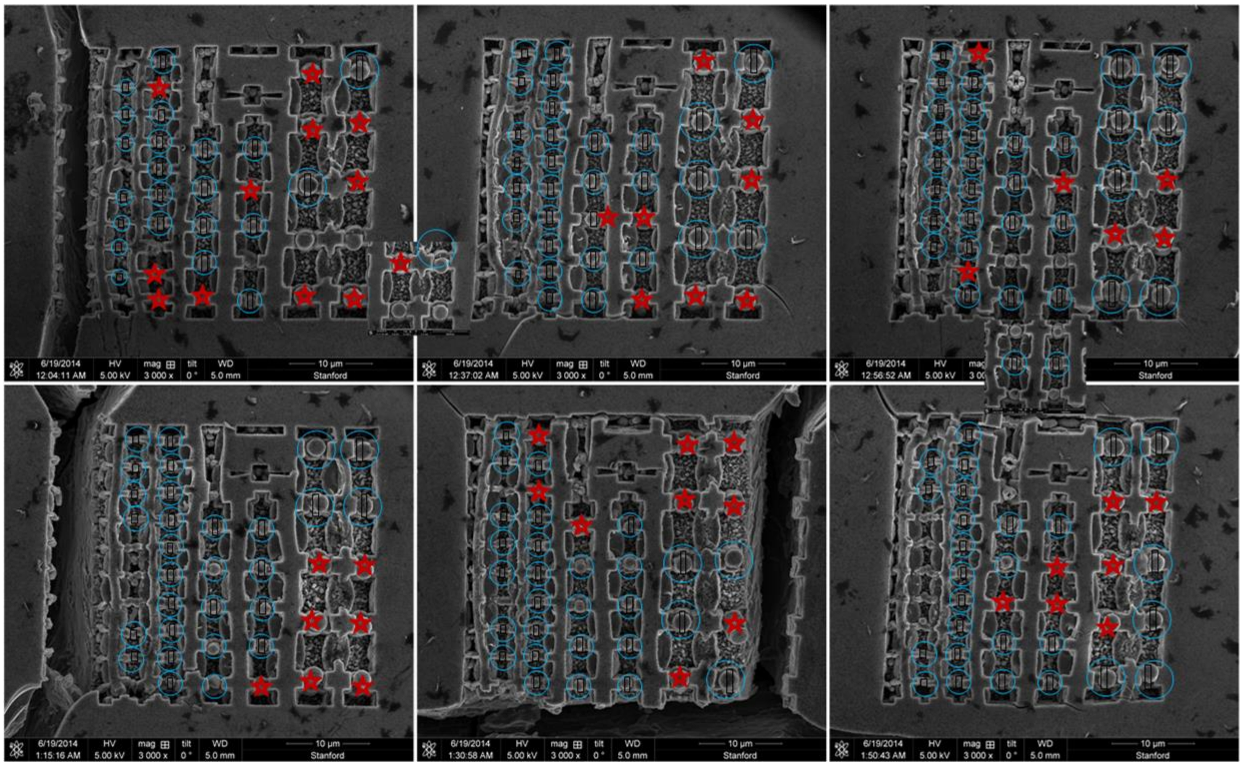

Supplementary Figure 4 | SEM images of the array of clamped Si pillars of various diameter after lithiation for statistic study of fracture ratio. Blue circle and red star indicate un-fractured and fractured pillars, respectively.

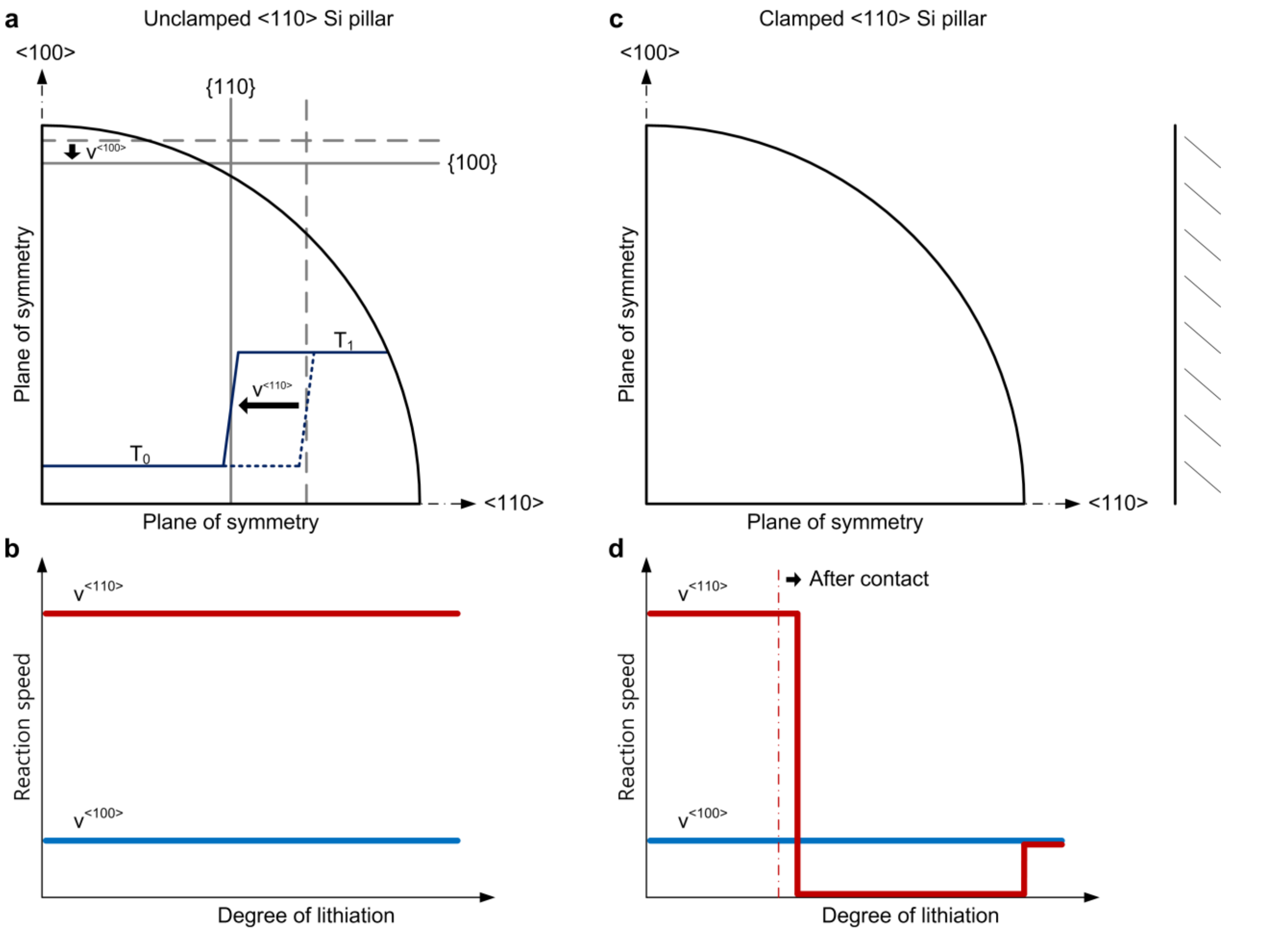

Supplementary Figure 5 | Schematic view of model description of numerical analysis of lithiation of unclamped and clamped <110> Si pillar. a, Geometry of simulated structure for unclamped case. The artificially defined temperature profile (dark blue line) represents Li concentration and propagates with predefined speed for each crystal orientation upon lithiation. **b,** Reaction speed defined as marching speed of artificial moving interface for the degree of lithiation when Si pillar is unclamped. The speed is constant. $\nu^{<110>} : \nu^{<110>} = 5 : 1$. **c,** Geometry of simulated structure with the rigid wall for clamped case. **d,** Reaction speed defined as marching speed of artificial moving interface for the degree of lithiation when Si pillar is clamped. After contact, $\nu^{<110>}$ is zero due to the stoppage of the reaction along <110> direction.

At the end of the lithiation, $\nu^{<110>}$ is same with $\nu^{<100>}$ assuming relaxation of compressive normal stress along <110> direction due to large expansion along <100>.

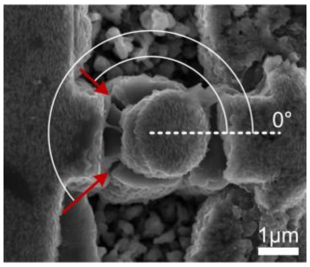

Supplementary Figure 6 | SEM image of fractured pillar explains how the location of the cracks on the pillar are measured. Center of the pillar and a reference line of the angle (dot line) are defined. Red arrows indicate the cracks and their angles are measured.

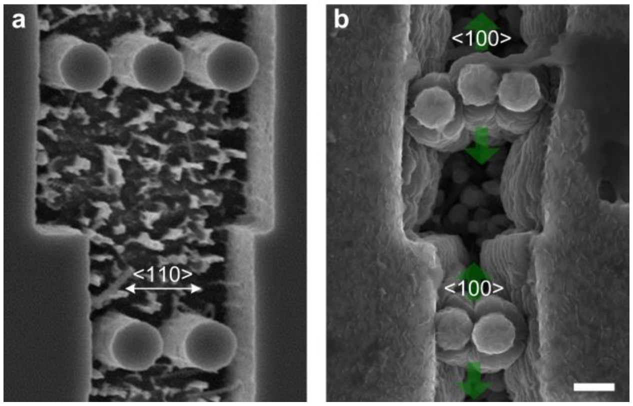

Supplementary Figure 7 | SEM study of the lithiation process of a mechanically clamped <110> Si nanopillar. a. Three and two pillars standing along <110> direction between rigid walls. The diameter of pillar is ~600 nm and the gap is ~250 nm. **b.** Lithiated pillars clamped along <110> direction showing the second preferential expansion along <100> direction. The scale bar is 500 nm.

Supplementary Table

Supplementary Table 1 | Mechanical properties of crystalline Si and Li_xSi used for analytical modeling and numerical analysis.

Material	Elastic		Plastic
	E [GPa]	ν	σ_Y [GPa]
Crystalline Si	185	0.28	7
Li_xSi	35	0.22	1

Supplementary Notes

Supplementary Note 1: Analytical model of normal stress at {110} interface upon the contact for the depth of lithiation

After contact of swelling Si structure, since the deformation is fully constrained by the contact and the interfacial compatibility, additional plastic deformation is no longer possible and additional lithiation induced strain must be accommodated by the elastic deformation. In this case, normal stress at {110} interface (σ_n) is applied by constraint of displacement to a half of the initial gap, *g/2* and defined as:

$$\sigma_n = E_{Si}\varepsilon_{Si} = E_{Li_xSi}\varepsilon_{Li_xSi} \tag{1}$$

where E_{Si} and E_{LixSi} are Young's modulus of crystalline Si and Li_xSi, respectively, and ε_{Si} and ε_{LixSi} are strain of crystalline Si and Li_xSi, respectively. The dimensions of the structures are defined in Fig. 3b in the main text. When silicon expands without constraint, the thickness of crystalline Si and Li_xSi are defined as t_{Si} and t_{LixSi}, respectively. When the whole structure is mechanically clamped by the contact, the thickness of deformed crystalline Si and Li_xSi are limited by a sum of the initial thickness of crystalline Si, t_0 and half of the initial gap, $g/2$. Then, the adjusted displacement of the whole structure due to the mechanical clamping is expressed as:

$$\Delta t = (t_{Si} + t_{Li_xSi}) - (t_0 + g/2) \tag{2}$$

Upon lithiation without constraint, t_0 is the initial thickness of crystalline Si, t_1 is the thickness of consumed crystalline Si and volume change of fully lithiated $Li_{3.75}Si$ is 400 % ($\Omega^{LixSi} = 4\Omega^{Si}$). Then,

$$t_{Si} = t_0 - t_1 \tag{3a}$$

$$t_{Li_xSi} = 4t_1 \tag{3b}$$

Apply equation (3) to (2). Then,

$$\Delta t = 3t_1 - g/2 \tag{4}$$

$$\varepsilon_{Si} t_{Si} + \varepsilon_{Li_xSi} t_{Li_xSi} = -\Delta t = -\left(3t_1 - \frac{g}{2}\right) \tag{5}$$

Apply equation (1) and (3) to (5). Then,

$$\frac{\sigma_n}{E_{Si}} t_{Si} + \frac{\sigma_n}{E_{Li_xSi}} t_{Li_xSi} = -\left(3t_1 - \frac{g}{2}\right) \tag{6}$$

Finally, normal stress of mechanical clamped lithiated Si after contact for the depth of lithiation (t_1/t_0) is expressed as:

$$\sigma_n = -\left[E_{Si}E_{Li_xSi}(3\frac{t_1}{t_0} - \frac{g}{2t_0})\right] \Big/ \left[E_{Li_xSi} + (4E_{Si} - E_{Li_xSi})\frac{t_1}{t_0}\right], \quad \text{when } 3\frac{t_1}{t_0} \geq \frac{g}{2t_0} \tag{7}$$

Supplementary Note 2: Finite element analysis of lithiation of unclamped/clamped Si nanopillars

Finite element package (ABAQUS, 2010 version) estimated stress evolution of Si nanopillar upon lithiation, with considering isotropic elastic and perfectly plastic model as our previous study presented[2]. The used mechanical properties are shown in Supplementary Table 1. We used the thermal expansion model of the lithiated Si for the given temperature field as an analogy of volume expansion for the concentration of Li in Si and calculated corresponding stress in the structure. To mimic the sharp interface of crystalline Si and Li_xSi moving toward the center upon lithiation, we defined artificial discrete temperature profile marching with given speed as shown in Supplementary Fig. 6a. Then, T_0 and T_1 represent zero Li concentration in crystalline Si and fully lithiated Si ($Li_{3.75}Si$) with 400% volume change (300% volume expansion), respectively. For the unclamped case of 550nm diameter <110> Si pillar, <110> direction (lateral) has

preferential expansion than <100> direction (vertical), so marching speed along <110> direction is defined 5 times faster than along <100> direction ($\nu^{<110>} : \nu^{<100>} = 5 : 1$) according to the experiment (Supplementary Fig. 6b). The clamped case has same structure and temperature profile with unclamped case before the contact (Supplementary Fig. 6c). But, lateral displacement is confined to 160 nm and $\nu^{<110>}$ is zero after the full contact as the experiment observed (Supplementary Fig. 6d). However, at the last stage of lithiation, compressive normal stress along <110> direction is relaxed due to large deformation along <100> direction, so the reaction front along<110> direction is set to move again with the same velocity as $\nu^{<100>}$. The simulation results show Li concentration profile and corresponding in-plane principle stress profile for both cases in Supplementary Movie 6 and 7, respectively.

Supplementary References

1. Limthongkul, P., Jang, Y.-I., Dudney, N. J. & Chiang, Y.-M. Electrochemically-driven solid-state amorphization in lithium-silicon alloys and implications for lithium storage. *Acta Mater.* **51,** 1103–1113 (2003).

2. Ryu, I., Lee, S. W., Gao, H., Cui, Y. & Nix, W. D. Microscopic model for fracture of crystalline Si nanopillars during lithiation. *J. Power Sources* **255,** 274–282 (2014).
